# Use of Tramadol or Other Analgesics in Patients Treated in the Emergency Department as a Risk Factor for Opioid Use

**DOI:** 10.1155/2020/8847777

**Published:** 2020-11-20

**Authors:** Jorge Enrique Machado-Alba, Laura Sofía Serna-Echeverri, Luis Fernando Valladales-Restrepo, Manuel Enrique Machado-Duque, Andrés Gaviria-Mendoza

**Affiliations:** ^1^Grupo de Investigación en Farmacoepidemiología y Farmacovigilancia, Universidad Tecnologica de Pereira-Audifarma S.A, Address: Calle 105 No. 14-140, Pereira, Risaralda, Colombia; ^2^Grupo de Investigación Biomedicina, Facultad de Medicina, Fundación Universitaria Autónoma de las Américas, Address: Ave Las Americas # 98-56, Pereira, Colombia

## Abstract

The objective of this cohort study was to determine the association between the use of tramadol in emergency departments and the later consumption of opioids at the outpatient level in a group of patients from Colombia. Based on a medication dispensation database, patients over 18 years of age treated in different clinics in Colombia who for the first time received tramadol, dipyrone, or a nonsteroidal anti-inflammatory drug (NSAID) in the emergency room between January and December 2018 were identified. Three mutually exclusive cohorts were created, and each patient was followed up for 12 months after the administration of the analgesic to identify new formulations of any opioid. A Cox proportional-hazards regression model was constructed to identify variables associated with receiving a new opioid. A total of 12,783 patients were identified: 6020 treated with dipyrone, 5309 treated with NSAIDs, and 1454 treated with tramadol. The mean age was 47.1 ± 20.4 years, and 61.6% were women. A total of 17.3% (*n* = 2207) of all patients received an opioid during follow-up. Those treated with tramadol received a new opioid with a higher frequency (*n* = 346, 23.8%) than the other cohorts (14.7% NSAIDs and 17.9% dipyrone, both *p* < 0.001). In the tramadol group, using more than 10 mg of morphine equivalents was associated with a greater use of new opioids (HR:1.47, 95%CI:1.12–1.93). Patients treated with tramadol in emergency departments have a higher risk of opioid use at the one-year follow-up than those treated with NSAIDs or dipyrone.

## 1. Introduction

Opioids are a group of drugs used mainly in the treatment of acute pain of moderate to severe intensity, as well as for the management of chronic pain, especially in cancer patients; however, they are not a first-line option in chronic noncancer pain [1, 2]. The continuous use of these medications generates tolerance, which can lead to abuse and physical and mental dependence, in addition to an increased risk of adverse drug reactions [3, 4]. Some estimates show that there are 15 million people in the world who are dependent on these drugs [5]. In the last two decades, the prescription of opioids has increased by 300% [6], and with it there has been an increase in cases of overdose and related deaths, due in part to their greater use in the treatment of chronic noncancer pain [5, 6]. According to the 2019 report of the United Nations Office on Drugs and Crime, it was estimated that 53.4 million people consumed opioids in 2017, 56% more than in 2016. A total of 29.2% consumed heroin or opium, which was 50% higher than the 2016 estimate [7].

On the other hand, Cicero et al. described that 47.1% of patients with substance-use disorders had their first exposure to opioids after a prescription by a doctor for pain management [8]. Numerous studies have shown that the use of opioids in the postoperative period increases the risk of their chronic use [5, 9–13]. Among the identified risk factors for chronic consumption of opioids are a history of alcohol or psychoactive substance abuse, current or previous smoking, comorbidities, psychiatric disorders, use of antidepressants or benzodiazepines, lower educational level, and lower income [5, 9–12]. Tramadol has been considered a moderately narcotic opioid analgesic since it is a partial agonist of *μ* (mu) receptors, and it has been found that it has a lower risk of dependence and other adverse reactions compared to other opioid analgesics, such as morphine, methadone, and fentanyl [4, 14, 15]. However, recently, in the USA, patients with postsurgical pain treated with tramadol were found to have a 6% greater risk of receiving an opioid again after 90 to 180 days compared to patients treated with other short-acting opioids [13].

In studies conducted in Colombia, codeine (57.7%) and tramadol (30.9%) were the most prescribed opioid analgesics in outpatients [16], whereas in patients with postoperative pain in eight clinics in the country, tramadol was the most commonly used (25.8%) [17]. Given the high frequency of the use of tramadol along with the little information about the real risk of increasing the probability of using opioids again, we sought to determine the association between the administration of tramadol or other nonopioid analgesics in emergency departments and the risk of later opioid consumption at an outpatient level in Colombian patients.

## 2. Materials and Methods

A cohort study was conducted with patients seen at five clinics in Colombia between January 1 and December 31, 2018, of either sex, older than 18 years, who received tramadol, nonsteroidal anti-inflammatory drugs (NSAIDs), or dipyrone during their stay. The patients were identified from the drug dispensing database of Audifarma SA, which delivers drugs to different clinics and hospitals in the country as well as to outpatients.

Three mutually exclusive follow-up cohorts were created depending on which drug they received (at least one dose) in the emergency department:

- *First cohort:* patients who were administered tramadol orally or parenterally during their stay.
- *Second cohort:* patients who were administered some NSAID or acetaminophen (which was included in the group of NSAIDs for their similar mechanism of action) orally or parenterally.
- *Third cohort:* patients who were administered dipyrone orally or parenterally.

The patients of the three cohorts were over 18 years of age, of any sex, and were affiliated with any of the healthcare insurance companies to which Audifarma SA dispensed medications, so that prior and subsequent prescriptions could be identified in the database that contains information on each subject and ensures that the patient is active in the Colombian health system.

### 2.1. Criteria for Exclusion

Patients who received an opioid, a benzodiazepine, or an antidepressant for up to 24 months before emergency room care at their respective clinics, either at prior visits to any of these services or at the outpatient level, were excluded from the three cohorts. Also excluded were those who received combined analgesia during their stay (a combination of tramadol and any other opioid, a NSAID, acetaminophen, or dipyrone or combinations of these). Similarly, people who during the 12-month follow-up period were administered an opioid for new hospitalization were excluded.

### 2.2. Variables

A database was created from the information stored on the medications dispensed to these patients, and the following variables were included:

- Sociodemographic: age, sex, and city of residence, grouped according to geographic region of the country, distributed as follows: 1- central-western region, 2- Atlantic region, 3- Bogota and eastern region, 4- southwestern region, and 5- southern region.
- Pharmacological: (a) received opioid: tramadol, morphine, fentanyl, codeine, etc.; (b) NSAIDs: diclofenac, ibuprofen, naproxen, etc.; (c) acetaminophen; and (d) dipyrone (metamizole sodium). The patients received doses even during the hospitalization beyond the stay in the emergency department). The doses were calculated according to the defined daily dose (DDD) (according to the World Health Organization, DDD is the mean dose used each day of a drug in its main indication in adults), and in the case of tramadol, its dosage was converted to morphine equivalents. Total morphine equivalents were calculated by multiplying the quantity of each prescription by the strength of the prescription (milligrams of opioid per unit dispensed). The quantity × strength product was then multiplied by the conversion factor for morphine equivalents (0.1 for tramadol) to estimate the morphine equivalents for the prescription.
- Comorbidities (sought from the codes of the International Classification of Diseases ICD-10 at the time of emergency room care) related to painful disorders or the use of drugs with addictive potential (cancer, anxiety, psychoactive substance use, alcoholism, depression, epilepsy, schizophrenia, bipolar affective disorder, depression, diabetes mellitus, migraine, other types of headaches, rheumatoid arthritis, ankylosing spondylitis, fibromyalgia, and osteoarthritis).

### 2.3. Outcome

Patients from each cohort were followed up for 12 months from the date they first received tramadol or the nonopioid analgesic to identify the new formulations of any opioid medications that they had received as outpatients. The time to prescription of the opioid, the duration of use (in days), the opioid medication received, and the dose were determined.

### 2.4. Statistical Analysis

The statistical package SPSS 26.0 for Windows (IBM, USA) was used to analyze the data. Frequencies, proportions, means, and standard deviations were established. For the comparison of the cohorts, parametric and nonparametric tests were used according to the normality of the distribution. Kaplan–Meier type analysis was used to establish the time until the prescription of a new opioid, where *T*_*o*_ was the index moment at which each patient first received the analgesic in the emergency department and *T*_*k*_ was the moment at which a new opioid was received. Bivariate analyses were performed to compare the different variables (age, sex, geographic region, dipyrone, NSAIDs, and comorbidities) identified based on whether the opioid was received or not during the 12 months after emergency care, using X^2^ tests. Cox proportional-hazards model was performed in which the dependent variable was receiving or not receiving a new opioid, and the independent variables were those identified as associated with the bivariate analyses. In addition, a subanalysis to calculate the risk of receiving a new opioid in the following 12 months according to the initial dose of tramadol was performed, as follows: low doses (1 to 9 mg morphine equivalents) or medium doses of tramadol (≥10 mg morphine equivalents). *p* < 0.05 was statistically significant.

### 2.5. Ethics Approval

The protocol was approved by the Bioethics Committee of the Universidad Tecnológica de Pereira in the risk-free research category. The ethical principles established by the Declaration of Helsinki were respected. No personal data of the patients were used.

## 3. Results

A total of 12,783 patients were identified in the emergency departments of five tertiary-care clinics in Colombia, affiliated with 11 different health insurers, who received one of the analgesics of interest. Dipyrone was the most frequent (*n* = 6020, 47.1%), followed by NSAIDs (*n* = 5309, 41.5%) and tramadol (*n* = 1454, 11.4%). The most frequent kind of dipyrone was injectable sodium at 1 g/2 mL (*n* = 3761, 62.5% of patients went with this analgesic), followed by dipyrone associated with hyoscine butyl bromide 2.5 g/20 mg (*n* = 2257, 37.5%). The most often prescribed NSAIDs were diclofenac sodium in an injectable solution of 75 mg/3 mL (*n* = 3360, 63.3%) and acetaminophen 500 mg tablets (*n* = 1880, 35.4%). The most widely used type of tramadol was an injectable solution of 50 mg/1 mL (*n* = 1393, 95.8%). The three cohorts differed in sex and age (see Table 1).

The majority of patients received the analgesic for only one day (*n* = 11,205, 87.7%, mode: 0; range: 0–18 days). A total of 82.7% (*n* = 4976) of the patients received dipyrone alone on the first day versus 88.9% (*n* = 1292) who received tramadol and 92.9% (*n* = 4936) NSAIDs, who on average received more doses of the drug (2.6 units/patient) compared with those of dipyrone (1.5 per patient) and tramadol (1.2 per patient). A total of 93.9% of the medications were prescribed by a general practitioner. The relationship between the mean dose and DDD showed that patients treated with dipyrone received 0.73 DDD, and those with diclofenac, acetaminophen, ibuprofen, and tramadol received 0.76 DDD, 0.89 DDD, 0.5 DDD, and 0.2 DDD, respectively.

### 3.1. Comorbidities

The most frequently found comorbidities were diabetes mellitus (*n* = 391, 3.1%), osteoarthritis (*n* = 188, 1.5%), cancer (*n* = 184, 1,4%), other types of headaches (*n* = 119, 0.9%), migraine (*n* = 108, 0.8%), depression (*n* = 99, 0.8%), anxiety (*n* = 97, 0.8%), epilepsy (*n* = 84, 0.7%), fibromyalgia (*n* = 61, *n* = 0.5%), rheumatoid arthritis (*n* = 58, 0.5%), schizophrenia (*n* = 24, 0.2%), bipolar affective disorder (*n* = 16, 0.1%), ankylosing spondylitis (*n* = 3, 0.0%), and psychoactive substance use (*n* = 2, 0,0%). Statistically significant differences in the bivariate analysis were found in tramadol versus dipyrone patients in terms of the frequency of suffering some comorbidities at the time of receiving care in the emergency room, especially cancer (tramadol 3.2% vs dipyrone 1.3%; *p* = 0.001), anxiety (1.8% vs 0.5%; *p* = 0.001), epilepsy (1.0% vs 0.5%; *p* = 0.038), diabetes mellitus (4.6% vs 2.7%; *p* = 0.001), and osteoarthritis (2.1% vs 1.3%; *p* = 0.12). Statistically significant differences in the bivariate analysis were also found when comparing patients with tramadol versus NSAIDs in terms of the frequency of some comorbidities such as cancer (tramadol 3.2% vs NSAIDs 1.1%; *p* = 0.001), anxiety (1.8% vs 0.8%; *p* = 0.001), and diabetes mellitus (4.6% vs 3.0%; *p* = 0.003).

### 3.2. Use of Opioids at One-Year Follow-Up

**Figure 1:**
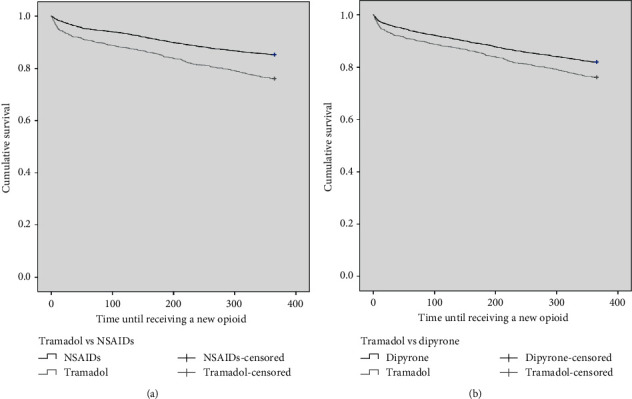
(a) Kaplan–Meier type analysis of the time until receiving a new opioid from the tramadol and NSAID patient cohorts at five clinics in Colombia, 2018-2019. (b) Kaplan–Meier type analysis of the time until receiving a new opioid from the tramadol and dipyrone patient cohorts at five clinics in Colombia, 2018-2019.

A total of 17.3% of all patients received an opioid in the 12 months after emergency care. Patients in the tramadol cohort received a new opioid during follow-up more frequently than those in the other cohorts, and in addition, the time before receiving any opioid was significantly less (Table 1 and Figures 1(a) and 1(b)). Approximately 85.9% of the new opioids were prescribed by general practitioners. The most frequently used opioids in the follow-up period were acetaminophen + codeine tablet 325 + 8 mg (*n* = 869), morphine injectable solution 10 mg (*n* = 334), dihydrocodeine syrup 12.1 mg (*n* = 283), fentanyl injectable solution 0.5 mg (*n* = 270), acetaminophen + hydrocodone tablet 325 + 5 mg (*n* = 161), meperidine injectable solution 100 mg, remifentanil injectable solution 2 mg (*n* = 115), oxycodone tablet 10 mg (*n* = 26), methadone tablet 40 mg (*n* = 8), and buprenorphine patch 20 mg (*n* = 6).

### 3.3. Cox Regression Comparing the Cohorts

**Figure 2:**
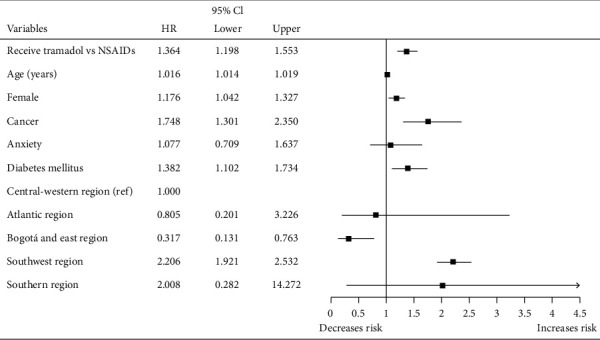
Cox regression of tramadol versus nonsteroidal anti-inflammatory drugs on the probability of receiving a new opioid up to 12 months of follow-up after being treated in emergency departments of five clinics in Colombia.

**Figure 3:**
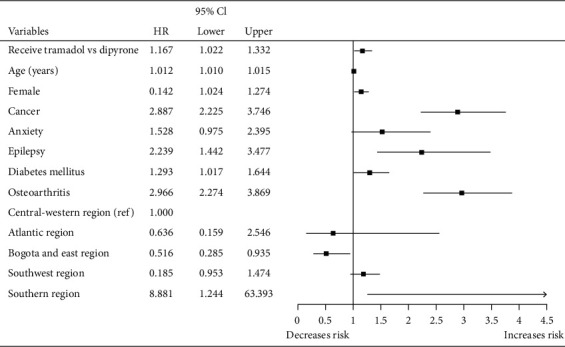
Cox regression of tramadol versus dipyrone on the probability of receiving a new opioid up to 12 months of follow-up after being treated in emergency departments of five clinics in Colombia, 2018-2019.

When comparing the cohort of patients who received tramadol with each of the other two cohorts (NSAIDs and dipyrone) by Cox regression adjusted for age, sex, and geographic area, we found that those who received tramadol were more likely to receive a new opioid in the following 12 months. In addition, a higher probability to receive a new opioid was found for each year of life gained, in women, patients with cancer, diabetes mellitus, epilepsy, osteoarthritis, and in patients served in the southwestern and southern regions of the country, while those seen in Bogota and the eastern region were less likely to receive one (Figures 2 and 3).

### 3.4. Analysis according to the Dose of Tramadol in Milligrams of Morphine Equivalents

When considering the dose of tramadol initially received (expressed in milligrams of morphine equivalents), we found that receiving a dose greater than or equal to 10 mg of morphine increased the risk of receiving an opioid again in the following 12 months (HR:1.47; 95%CI:1.12–1.93), as did increasing age (HR:1.01; 95%CI:1.00–1.01) and having received the first opioid in a clinic in a region other than the central-western region of the country (HR:1.57; 95%CI:1.23–2.00).

## 4. Discussion

The present study found an association between the use of tramadol in emergency departments and the risk of receiving an opioid again in the 12 months after its prescription compared to the use of NSAIDs, acetaminophen, or dipyrone in a group of patients aged over 18 years, affiliated with the Colombian health system. The mean age (53.1 years) of the cohort of patients who received opioids in this study was higher than that found in other studies (21.8–39.7 years) [18, 19], and the proportion of women (56.9%) was higher than that of men, which is consistent with what has been reported in other studies (52.5–72.3%) [5, 13, 18–20].

A total of 23.8% of the patients in the tramadol cohort received an opioid again in the 12 months after the initial exposure, which has also been evidenced in other studies [5, 19, 21–25]. In the USA, Hooten et al. found, in patients who had initially received an opioid who were followed up for one year, that 21% of them progressed to episodic prescriptions of opioids and 6% to continuous use [5]. Meisel et al., in patients who attended the emergency department and who were managed with opioids, found that 13.7% had persistent use [25]. Similarly, in several studies involving patients with nonobstetric postsurgical pain, frequencies of opioid use of 7.7–8.1% were found at one year after the initial exposure [21, 22], while in patients who underwent a cesarean section or hysterectomy, the persistence of opioid use was 0.3% [23] or 0.5% [24], respectively. In patients at a dental clinic, 6.9% received a new opioid between 90 and 365 days after the initial contact [19]. These data show a worrisome reality regarding the use of opioids in emergency departments and add robustness to the findings of the present study.

In recent years, multiple studies have demonstrated that there is an increased risk of the persistent use of opioids when they are used in the management of acute pain in patients who had not previously used them [13, 18, 22]. In Canada (OR:1.44; 95%CI:1.39–1.50) [22] and in the USA (OR:4.90; 95%CI:3.22–7.45), the risk was significantly higher [18]. These findings are consistent with the findings in our cohort of patients. However, in some studies, the risk of receiving an opioid again after an initial exposure has been low [13], and the number of patients who may be exposed to them has been very large due to the high prevalence of use, including tramadol [16, 17, 26], which has also increased in recent years [26].

Tramadol is a synthetic opioid, and its mechanism of action involves inhibiting the reuptake of norepinephrine and serotonin, in addition to being a partial agonist of the *µ* receptors [27, 28]; therefore, it has typically been considered an analgesic with little potential for dependence, compared to other short-acting opioids [29]. However, O-desmethyltramadol, its main metabolite generated in the liver, has a 700-fold-higher affinity for the *µ* receptor [27, 28], which can help to elucidate the apparent risk if patients will use it chronically. Some authors suggest that tramadol should be reclassified [13] since it is currently in category IV of the Controlled Substances Act (Scheduled IV), which refers to drugs with a low potential for abuse and dependence [29].

Risk factors for the persistent use of opioids after the initial contact include age, sex, preoperative pain, medical comorbidities, history of substance abuse, alcohol or tobacco use, low socioeconomic status, use of benzodiazepines or antidepressants, and the dose and duration of opioid use [7, 13, 19, 23–25]. We found that women were more likely to receive opioids than other drugs (13–15% more likely than NSAIDs or dipyrone), which was consistent with, but smaller in magnitude, the data found by Thiels et al. in the USA in postsurgical patients (OR:1.22; 95%CI:1.18–1.25) [13]. These data are also in line with those of Schroeder et al. in patients who required analgesic management in a dental clinic (OR:1.20; 95%CI:1.00–1.40; *p* = 0.01) [19]. The greater risk reported in women may be because they are among the population groups that most use the health services [30].

In addition, we found that the risk increased for each year of life, which was consistent with what was described by Swenson et al. in the USA (OR:2.75; 95%CI:1.86–4.06) [24]. Likewise, an initial dose of tramadol of 10 mg of morphine equivalents or more increased the risk of receiving an opioid as an outpatient, a trend that was also observed in other studies, but with higher tramadol doses [7, 13, 25], although other research has found no statistically significant differences [23].

The finding that cancer patients are more likely to receive opioids has already been documented, since tumors may need more effective analgesic management and they are more likely to require them at the time of emergency care, or after surgery, in the following year [31, 32]. The association found in patients with diabetes mellitus may be because they also suffer from diabetic neuropathy, which results in analgesia, paresthesia, and pain, which could yield to specific analgesics and eventually to opioids, particularly tramadol [33]. Osteoarthritis is a chronic pain disorder, which may eventually require, depending on the intensity of the symptom, analgesics capable of relieving pain when acetaminophen or NSAIDs fail to do so [34, 35]. Furthermore, it has already been observed that epilepsy can be a predictor of subsequent opioid use, particularly in patients with a painful knee and hip disorders [35].

Certain limitations should be considered when interpreting these results. There was no access to clinical records to verify the indications for the use of the different analgesics included in the study. Other covariates that may have influenced the decision of the physician on the initial management of emergencies with analgesics were not known. For these reasons, it is important to recognize the possibility of residual confounders in the findings. In addition, it is not possible to determine if patients purchased tramadol, which was formulated outside the health system. Other variables that could be associated with the consumption of psychoactive substances by patients, as well as their tobacco use, body mass index, income level, and educational level were also not identified.

## 5. Conclusions

Based on these findings, we can conclude that patients treated with tramadol in the emergency departments of some clinics in Colombia have a higher risk of opioid use during the 12 months following their care in comparison to those given NSAIDs or dipyrone. Predominating among the higher-risk group were patients of older age, women, and those receiving tramadol doses greater than 10 mg of morphine equivalents. Physicians should be cautious when prescribing tramadol and use it only when it is strictly necessary, and at the lowest effective doses. It is necessary to implement measures to educate prescribers about the risks of using tramadol and other opioids.

## Figures and Tables

**Table 1:** Clinical characteristics related to the prescription of tramadol, nonsteroidal anti-inflammatory drugs, and dipyrone in 12,783 patients treated in the emergency departments of five clinics in Colombia, 2018-2019.

Characteristics	Total	Tramadol	NSAIDs	Dipyrone	*p*
	12783	*n* = 1454	*n* = 5309	*n* = 6020	
Women	7872 (61.6%)	828 (56.9%)	3238 (61.0%)	3806 (63.2%)	<0.001
Age (years)	47.1 ± 20.4	53.1 ± 20.3	43.5 ± 19.5	48.8 ± 20.6	0.005
Mean doses ± SD (mg)		60.0 ± 29.7	Ds: 75.9 ± 10.1	1500 ± 1100	
			Ac: 2689 ± 2339		
Received opioid at follow-up	2207 (17.3%)	346 (23.8%)	778 (14.7%)	1083 (17.9%)	<0.001
Time to opioid in days		135.2 ± 114.7	143.1 ± 106.6	142.2 ± 110.3	<0.001
Range of time to receive opioid (days)	1–360	1–358	1–360	1–360	

NSAIDs: nonsteroidal anti-inflammatory; SD: standard deviation; Ds: diclofenac sodium; Ac: acetaminophen.

## Data Availability

The data used to support the findings of the study is included within the article.
